# United States: law and policy concerning transfer of genomic data to third countries

**DOI:** 10.1007/s00439-018-1917-9

**Published:** 2018-08-01

**Authors:** Mary Anderlik Majumder

**Affiliations:** Center for Medical Ethics and Health Policy, Baylor College of Medicine, One Baylor Plaza, Houston, TX 77030 USA

## Abstract

This paper provides an overview of US laws and related guidance documents affecting transfer of genomic data to third countries, addressing the domains of consent, privacy, security, compatible processing/adequacy, and oversight. In general, US laws governing research and disclosure and use of data generated within the health care system do not impose different requirements on transfers to researchers and service providers based in third countries compared with US-based researchers or service providers. Of note, the US lacks a comprehensive data protection regime. Data protections are piecemeal, spread across bodies of law that target specific kinds of research or data generated or held by specific kinds of actors involved in the delivery of health care. Oversight is also distributed across a range of bodies, including institutional review boards and data access committees. The conclusion to this paper examines future directions in US law and policy, including proposals for more comprehensive protections for personal data.

## Introduction

There is ample evidence of US government support for global data sharing to advance biomedical research and improve human health. Collins and Varmus (2015), leaders of the US National Institutes of Health (NIH) and National Cancer Institute, respectively, laid out their vision of a US Precision Medicine Initiative that would tap “the world’s brightest scientific and clinical minds” for insights and analysis and cooperate with similar projects in other countries (2015). Highlighting the gains from such collaborations, they implicitly invoked a history of US partnerships with research sponsors and scientists in other countries that has included the Human Genome Project and the International HapMap Project. At the end of 2017, the US Congress passed the 21st Century Cures Act (Act), which among other things, expressed Congressional support for NIH encouragement and facilitation of a global pediatric clinical study network (2016).

At the same time, US surveys provide evidence of public concern about transfer of data about US persons to researchers in third countries. In 2016, we conducted a US public opinion survey that found a 20% increase in discomfort with sharing health data when the recipients are specified as academic researchers outside of the US versus domestic researchers (Majumder et al. 2016). Our findings were consistent with the results of prior US surveys. Furthermore, we found that non-US researchers faced heightened distrust relative to the US-based researchers in relation to both privacy and security.

This paper provides an overview of US laws and related guidance documents affecting transfer of genomic data to third countries, addressing the domains of consent, privacy, security, compatible processing/adequacy, and oversight. In particular, two scenarios guide the analysis. First, a US-based researcher (Researcher A) contemplates sharing genomic data with a researcher in a third country (Researcher B). Is such data sharing permitted, and if yes, are there any special requirements that Researcher A must comply with owing to Researcher B’s location “offshore”? Second, a US-based health care provider contemplates sending genomic data to an entity in a third country for processing, interpretation, or provision of some other service. Are there any special requirements owing to the service provider’s location offshore? As explored in greater depth below, in general, US laws governing research and disclosure and use of data generated within the health care system do not impose different requirements on transfers to researchers and service providers based in third countries compared with US-based researchers or service providers. The conclusion examines future directions in US law and policy.

## Consent

In the US, there is no general or comprehensive data protection regime. The two bodies of law of greatest relevance to sharing of genomic data for research- and health care-related purposes are the Federal Policy for the Protection of Human Subjects (known as the “Common Rule”)^1^ and the Health Insurance Portability and Accountability Act (HIPAA 1996).

The Common Rule governs research conducted, supported, or otherwise subject to regulation by the US Department of Health and Human Services (DHHS), which encompasses NIH, and 16 other federal departments and agencies. In January of 2017, these departments and agencies published a Final Rule making extensive changes to the Common Rule (DHHS 2017). The initial general compliance date for the revised Common Rule was January 19, 2018. However, the general compliance date has been delayed until January 21, 2019 (DHHS 2018a, b). Still, since the rationale offered for this delay is giving the research community more time for implementation, there appears to be little doubt that the changes introduced by the Final Rule will eventually take effect. Hence, references to the Common Rule below are references to the Final Rule.

A study in which a researcher collects a biospecimen from an individual and carries out genotyping will be covered by the Common Rule, provided there is the proper nexus with 1 of the 17 federal departments and agencies. For data-focused research to fall within the scope of the Common Rule, a researcher must either gather the data by intervening or interacting with an individual or obtain, use, study, analyze, or generate “identifiable private information.” Information is “identifiable” if “the identity of the subject is or may readily be ascertained by the investigator or associated with the information” [DHHS 2017; to be codified at 45 CFR § 46.102(e)(5)]. Historically, genomic data with direct identifiers removed has not been considered identifiable, and sharing of genomic data has occurred without the consent of the individual from whom the data was derived (NIH 2014). In recent years, policy has moved in the direction of requiring consent before genomic data can be shared, but at the same time legitimizing broad consent (i.e., consent to research uses and users described in broad terms with a long or indefinite term absent withdrawal) and recognizing exceptions to consent requirements in special circumstances [see, for example, the NIH Genomic Data Sharing Policy (NIH 2014)]. Sensitivity to re-identification risk in the case of genetic sequence information has been one of the drivers of this policy shift.

The Common Rule does not treat genetic sequence information as by its nature identifiable. Instead, it directs departments and agencies to consult with experts (including experts in data matching and re-identification) to reexamine the meaning of “identifiable private information” within a year and at least every 4 years thereafter. In light of this reexamination, “[i]f appropriate and permitted by law,” the interpretation of the term may be altered, e.g., through the issuance of guidance [DHHS 2017; to be codified at 45 CFR § 46.102(e)(7)]. Moreover, experts are to be enlisted in assessing whether there are analytic technologies or techniques that should be considered to generate identifiable private information, on the same timetable. The preamble to the Final Rule indicates that whole genome sequencing will be the first technology reviewed under this provision.

Assuming that the Common Rule applies, it is unclear whether information about the possibility that data may be shared with researchers in third countries must be provided as part of the informed consent process. The Common Rule contains no special requirements for sharing of identifiable private information (or biospecimens, for that matter) with researchers in third countries, nor does it explicitly address such a scenario. The general standard for provision of information under the Common Rule is what “a reasonable person would want to have in order to make an informed decision about whether to participate” [DHHS 2017; to be codified at 45 CFR § 46.116(a)(4)]. In the case of broad consent encompassing sharing of identifiable private information with other researchers, potential participants must be provided with a description of “the types of institutions or researchers that might conduct research” [DHHS 2017; to be codified at 45 CFR § 46.116(d)(3)]. Based on our survey findings, for roughly a quarter of the US public, sharing with researchers in third countries heightens concerns about privacy and security (Majumder et al. 2016). Hence, information about international data sharing is potentially relevant to decision-making about participation.

The National Human Genome Research Institute (NHGRI 2018) webpages offering guidance and sample language for informed consent forms do not mention international data sharing (2018). The NHGRI website also includes a page providing access to sample consent forms used in NIH-funded research projects, with a disclaimer that they are not provided as guidance or templates. The sample informed consent form developed by the Electronic Medical Records and Genomics (eMERGE 2009) Network Consent and Community Consultation Workgroup Informed Consent Task Force includes a statement about access to data by non-US researchers. In context, the statement reads as follows: “Researchers can ask to study the materials stored in the Biobank. This includes researchers from [institution], as well as from other universities, the government, and drug- or health-related companies. Some researchers will be from the US, *some may be from other countries around the world*.” (emphasis added) (2009).

Interestingly, the Chair of the eMERGE Task Force, Laura Beskow, also led a Delphi study to determine which informational elements must be understood for a consent to be valid. For the topic of access to biospecimens/data, less than one quarter of the Delphi panelists considered it essential for prospective research participants to understand that researchers gaining access could potentially come “from all over the world” (Beskow et al. 2015). Even if disclosure of the possibility of international sharing is required because it is arguably information a reasonable person would want and/or an essential part of a description of the types of institutions or researchers that might conduct research, there is no requirement that individuals be offered a choice regarding international sharing. Indeed, unlike “Not-for-profit use only,” “Domestic use only” is not even on the list of Standard Data Use Limitations published by the NIH (Office of Science Policy 2018).

Regulations issued by the HHS Office for Civil Rights (OCR) under HIPAA apply only to “covered entities” and, to a more limited extent, their “business associates” (OCR 2002, 2003, 2009, 2013). This means protections do not attach to all personal health-related information created or circulating within the US; rather, protection depends on the status of the data holder. There are three categories of covered entities: health care providers (if they transmit electronic information in connection with transactions for which DHHS has adopted a standard), health plans (i.e., private health insurance companies, HMOs, company health plans, and government programs that pay for health care), and health care clearinghouses that process or exchange information. There are many entities that may hold personal health-related information that are neither covered entities nor business associates of covered entities and so operate outside HIPAA (e.g., wearables vendors like FitBit, technology companies like Apple, Facebook, and Google). Information also falls outside HIPAA if it is not “protected health information” (PHI), individually identifiable health information created or received by a covered entity (and not covered by one of four exclusions, such as for educational records protected by another federal law and persons deceased more than 50 years). Note that researchers that do not engage in the provision of health care may nonetheless be regulated by HIPAA if they work with PHI within an institution that is a covered entity. Finally, another part of the basic structure is that either of the following is sufficient for de-identification: (1) an expert applying generally accepted statistical and scientific principles and methods “determines that the risk is very small that the information could be used, alone or in combination with other reasonably available information, by an anticipated recipient to identify an individual” or (2) 18 types of identifiers are removed and the covered entity lacks “actual knowledge that the information could be used alone or in combination with other information to identify an individual” (45 CFR §§ 160.103, 164.514; OCR 2012).

One set of HIPAA regulations addresses privacy and is commonly referred to as the Privacy Rule (OCR 2002). Assuming the Privacy Rule applies, there are three main paths by which PHI can legally be shared: (1) with individual authorization, (2) with documented institutional review board (IRB) or privacy board approval, and (3) as a limited data set with specified direct identifiers removed subject to a data use agreement. Requirements for a valid authorization include the “name or other specific identification of the person(s), or class of persons, to whom the covered entity may make the requested use or disclosure” (45 CFR § 164.508). As with the Common Rule, the Privacy Rule does not explicitly address transfer of PHI to a researcher or service provider in a third country. To the extent international issues have drawn the attention of commentators, the commentary has focused on whether PHI collected from foreign nationals outside the US by researchers affiliated with covered entities is subject to HIPAA requirements (Prentice et al. 2004).

Finally, it is worth noting that one additional regulator, the Federal Trade Commission (FTC), is charged with protecting consumers from deceptive or unfair acts or practices in or affecting commerce. This has sometimes led the agency to act in the biomedical space, when it concludes that companies are misleading consumers about what is happening with their health information (Rich et al. 2016). However, at this point in time, there is no indication that the FTC is contemplating regulatory action with respect to consent processes related to the flow of genomic data to third countries, or other aspects of such transactions. The role of the FTC with respect to the Privacy Shield Framework, which governs the flow of data from the European Union (EU) and certain other countries to the US, is discussed below under “Compatible Processing/Adequacy.”

## Privacy

As noted above, in general, US laws such as the Common Rule and HIPAA do not prohibit sharing of genomic data with third countries nor do they create additional barriers to sharing genomic data with third countries, e.g., by imposing special requirements on researchers or others proposing to share genomic data with offshore individuals or entities. There are at least two possible qualifications to this statement. First, the Act contains a provision that might be interpreted to restrict the transfer of genomic data to third countries. Specifically, the Act includes provisions that strengthen the protections available under certificates of confidentiality (Certificates) issued by the NIH and its sister agencies. Historically, investigators who received federal funding to conduct research considered sensitive could choose to apply for a Certificate, which enabled them to refuse to disclose identifying characteristics of research participants in legal proceedings if they did not wish to do so.

Section 2012 of the Act directed the Secretary of DHHS to issue Certificates to researchers who receive federal funding automatically, as well as permitting issuance of Certificates to non-federally funded investigators upon application. Investigators covered by Certificates are prohibited from disclosing “identifiable, sensitive information” created or compiled in the course of the research “for perpetuity,” with a few exceptions. The Act does not define “sensitive” and sets a relatively low threshold for identifiability: if there is “at least a very small risk” that an individual’s identity could be deduced from the sum of available data using current scientific practices or statistical methods, then the information would be covered by the Certificate [42 USC. § 241(d)(4)].

One of the exceptions is for disclosures “made for the purposes of other scientific research that is in compliance with applicable Federal regulations governing the protection of human subjects in research” [42 USC. § 241(d)(1)(C)(iv)]. If federal human subjects research regulations are not applicable, as in the case of research carried out abroad without a nexus to a federal department or agency, the exception might be interpreted to permit disclosure if the intent behind the provision is simply to ensure that studies subject to federal research regulations like the Common Rule are in compliance with those regulations as a condition of disclosure. However, this interpretation has worrisome implications, since it means that no conditions restrict the transfer of information for purposes of research outside the scope of federal research regulations, including unregulated domestic research without oversight to ensure protection of privacy and security as well as regulated or unregulated offshore research. Alternatively, the provision could mean that only research governed by and in compliance with federal human subjects regulations qualifies for the research exception. In that case, much research in third countries as well as unregulated domestic research would be outside the exception’s scope. Note that the transfer would still be permitted with the consent of the individual, as that is a separate exception.^2^

Second, there have been discussions of the special care that may be required under the HIPAA Privacy and Security Rules when PHI is transferred offshore outside the regulations. The Office of the National Coordinator for Health Information Technology (ONC) has developed a *Guide to Privacy and Security of Electronic Health Information*. In a table of examples of potential information security risks with different types of EHR hosts, the Guide notes that with cloud-based EHRs, “data may be stored outside the US, and other countries may have different health information privacy and security laws that may apply to such offshore data” (ONC 2015). Possible mitigation steps include confirming that the EHR host follows US security standards and requirements. Some commentators advise analyzing the relevant laws and regulations in the third country to ensure that they are at least as protective as those in the US and conducting regular audits of offshore contractors’ activities. Because HIPAA does not have explicit extra-territorial reach, some have questioned whether DHHS would have jurisdiction to bring enforcement actions abroad in the event of a violation (Milliard 2016; McGee 2013).

## Security

In the current environment, even the most robust privacy protections for data in electronic form are essentially meaningless in the absence of security measures. The HIPAA Security Rule operationalizes the protections contained in the Privacy Rule by directing covered entities to establish reasonable and appropriate administrative, technical, and physical safeguards for PHI in electronic form (e-PHI) (OCR 2003). This includes ensuring the confidentiality, integrity, and availability for legitimate use of e-PHI, identifying and protecting against anticipated threats and impermissible uses or disclosures, and taking steps to ensure compliance by their workforce. The emphasis in the Security Rule is on flexibility and scalability, given the diversity of covered entities, as well technology neutrality, given the rapid pace of technological evolution. Covered entities are permitted to, indeed must, consider their size, complexity, and capabilities, their infrastructure, the costs of security measures, and the likelihood and possible impact of potential risks, and they must review and modify their security measures over time.

Under the Security Rule, some implementation specifications for standards are required (must be implemented) while others are addressable (subject to a determination by the covered entity or business associate regarding whether the implementation specification is reasonable and appropriate in its environment; if it is not, alternative measures must be adopted if reasonable and appropriate). For example, within the technical safeguards section, the data integrity standard mandates implementation of policies and procedures to protect e-PHI from improper alteration or destruction; the implementation of electronic mechanisms to corroborate that e-PHI has not been subject to unauthorized alteration or destruction is addressable. Likewise, the transmission security standard mandates implementation of technical security measures to guard against unauthorized access to e-PHI transmitted over an electronic connection network; the implementation of an encryption mechanism is addressable.

Covered entities and their business associates are also subject to the HIPAA Breach Notification Rule (OCR 2009). All cyber-security incidents involving access, acquisition, use, or disclosure of PHI are subject to the notification requirements unless the information was encrypted and the relevant actor determines, through a written risk assessment, that there is a low probability that the PHI was compromised. If the breach affected 500 or more individuals, notice must be given to the OCR as soon as possible but no later than 60 days after discovery, and notice must also be given to the affected individuals and the media (unless a law enforcement official requests a delay). Such breaches are made public via what is often referred to as the “Wall of Shame” on the OCR web page (OCR 2018). If fewer than 500 individuals are affected, notice must still be given to OCR and affected individuals, but within a longer timeframe. The FTC has implemented similar requirements for vendors of personal health records and their third-party service providers (FTC 2009).

Risk analysis and management fall under the heading of administrative safeguards, and this seems the most likely legal prompt for covered entities to consider and manage any increases in risk associated with transfers to third countries. The Security Rule itself does not discuss offshore transfers. But, as noted above, a guidance document published by the ONC discusses risks associated with use of cloud-based services that may involve offshore transfers and suggests a mitigation step of confirming compliance with US security standards and requirements. Furthermore, commentators stress the importance of using contracts to manage risk, while recognizing the potential enforcement challenges (Milliard 2016; Dove et al. 2015; McGee 2013). For example, US based genomic researchers might restrict their search for a cloud service provider to entities that hold “trusted partner” status or are able and willing to sign a HIPAA “business associate agreement” and have current third-party audit certifications (Dove et al. 2015).

## Compatible processing/adequacy

Neither the Common Rule nor HIPAA uses the concept of “compatible processing” to capture an assessment of data protection regimes in other countries as a condition to the transfer of data. Any such assessment would be carried out as part of a general risk assessment and risk mitigation strategy, as described above, or as a step required for an IRB, data access committee (DAC), or privacy board determination regarding the adequacy of provisions to protect subject privacy, as described below.

“Adequacy” is important in determining whether personal data can flow from the EU, Norway, Liechtenstein, and Iceland to the US without special safeguards. (Switzerland has similar standards, and other countries outside the EU may use EU protections as a benchmark.) The current EU-US Privacy Shield Framework is the subject of an affirmative adequacy decision by the European Commission dated July 12, 2016. The adequacy decision references a ruling by the European Court of Justice that an adequate level of protection does not necessitate protections that are identical to those guaranteed in the EU legal order. It must, however, ensure a level of protection of fundamental rights and freedoms that is “essentially equivalent” to the EU level and proves effective in practice (European Commission 2016). The Privacy Shield Framework, which is administered by the US Department of Commerce, sets up a self-certification process under which eligible organizations publicly commit to comply with notice, data integrity and purpose limitation, choice, security, access, recourse, enforcement and liability, and accountability for onward transfer principles. The process is voluntary, but once an organization makes these commitments, they become enforceable against the organization by the FTC (note that the FTC does not have jurisdiction over most non-profit organizations).

The Privacy Shield Framework undergoes annual reviews, and there are signs of trouble in connection with the upcoming review. These include a resolution from the European Parliament taking the view that the current arrangement does not provide an adequate level of protection and calling for its suspension on September 1, 2018 unless full compliance is achieved, and a case that may bring scrutiny from the European Court of Justice, which struck down the prior Safe Harbor Framework (Lomas 2018).

## Oversight

IRBs have primary responsibility for oversight under the Common Rule. While some research is entirely outside the scope of the Common Rule (for example, research with non-identifiable information) or entirely exempt from IRB review (for example, secondary research with identifiable private information that is publicly available), the revised Common Rule mandates “limited IRB review” as a condition for some exemptions, including the exemption for secondary research within the parameters of a broad consent [DHHS 2017; to be codified at 45 CFR § 46.104(d)(8)]. “Secondary research” is not defined in the regulations. However, the preamble to the Final Rule describes secondary research as “re-using identifiable information and identifiable biospecimens that are collected for some other ‘primary’ or ‘initial’ activity” (DHHS 2017, p. 7191). There is no requirement under the Final Rule that the information and biospecimens be pre-existing at the time the secondary research study begins, as was the case under the earlier version of the Common Rule.

In the case of secondary use pursuant to broad consent, limited IRB review includes making and documenting a determination that there are “adequate provisions to protect the privacy of subjects and to maintain the confidentiality of data” [DHHS 2017; to be codified at 45 CFR § 46.111(a)(7)]. The Secretary of DHHS is charged with issuing guidance concerning this requirement, but such guidance is not yet available. The IRB must also confirm that the research to be conducted is within the scope of the broad consent. Finally, the study plan cannot include returning individual research results to subjects, although this does not prevent an investigator from abiding by any legal requirements to return individual research results.

IRBs also make the determinations that may lead to waiver (or alteration) of consent, which could be relevant in the case of a proposed secondary use in the absence of broad consent. When considering a request for waiver of consent in the context of research with data, the IRB must find that the research involves no more than minimal risk and could not practicably be carried out without the requested waiver and without using information in an identifiable format, and that the waiver will not adversely affect the rights and welfare of subjects. The word “practicably” is not defined in the regulations. The preamble to the Final Rule does note that the Secretary’s Advisory Committee on Human Research Protections recommended that “this requirement be interpreted to mean that it would be impracticable to perform the research, not impracticable to obtain consent due to financial or administrative burdens, without the waiver” (DHHS 2017, p. 7225).

None of these provisions describing the role of the IRB in oversight distinguish between domestic secondary research and secondary research conducted in third countries. DACs are an additional mechanism for oversight of data, including genomic data. For example, the DAC structure for the database of Genotypes and Phenotypes (dbGaP), a public repository created by the US National Center for Biotechnology Information, is described in its Certificate:

To protect participants’ confidentiality, there will be on-going assurance of participant protections pertaining to data held within dbGaP based on oversight by the NIH of data access to ‘secondary’ data users. Research access to dbGaP data will be provided through a ‘Controlled Access’ process implemented by NIH Data Access Committees (DACs). Data Access Committees will be constituted by the NIH Institutes with federal employees possessing the appropriate scientific and bioethics expertise, and through the oversight and actions of these committees access to dbGaP datasets will be provided based on the consistency of specific research uses (proposed by data requestors) with the data use limitations set by the institutions submitting the datasets to the NIH. Approved data users will agree, along with their home institutions, to follow specified principles and terms of use for the specific dataset provided. NIH will monitor data use practices over time to assure that policies and procedures for protecting participants and their interests remain robust (Confidentiality Certificate 2008).

Access to individual-level data housed in dbGaP is under the jurisdiction of the sponsoring institute, as identified on the study report page. The application for access or “Data Use Certification” must include the controlled dataset(s) to which the researcher is seeking access, a description of the proposed research use, and assurances that the data will only be used for approved research and will not be sold or shared with third parties, confidentiality will be protected, all applicable laws, policies, and terms of use will be followed, and no attempts will be made to re-identify study participants, contributing investigators and funders will be acknowledged in publications, and data and conclusions derived from them will remain in the public domain, and annual research reports will be submitted to the relevant DAC. In the case of applications from non-NIH researchers, the principal investigator must be a tenure-track professor, senior scientist, or equivalent and complete a registration process (NIH 2018). Relevant here is the fact that dbGaP houses datasets generated in third countries and shares data with researchers in third countries, and there are no special standards for sharing with non-US researchers.

Turning to HIPAA, under the HIPAA Privacy Rule and Security Rules covered entities must designate a privacy official (sometimes referred to as the “privacy officer”) and a security official, respectively, with responsibility for the development and implementation of policies and procedures to ensure compliance (OCR 2002, 2003). While it is fairly common for an expert in information technology to be assigned the role of security official, as the discussion above suggests, the role includes identifying and managing risk through training and contracting as well as implementation of technical safeguards. These aspects of the position require an awareness of possible vulnerabilities related to international transactions.

Covered entities may also establish privacy boards to act upon requests for waiver (or alteration) of authorization requirements under the Privacy Rule in lieu of an IRB. To approve a waiver request, a privacy board or IRB must determine that: (1) the use or disclosure of PHI involves no more than a minimal risk to the privacy of individuals, based on, at least, the presence of an adequate plan to protect the identifiers from improper use and disclosure, destroy them at the earliest opportunity (consistent with the conduct of the research, health and research considerations, and legal requirements), and adequate written assurances that the PHI will not be reused or redisclosed (except as required by law, for authorized research oversight, or for other permitted research); (2) the research could not practicably be conducted without the waiver or alteration; and (3) the research could not practicably be conducted without access to and use of the PHI (OCR 2002). Again, there is no discussion of special standards for research conducted by researchers based in third countries.

## Future directions

There have been many twists and turns in the story of US foreign and trade policy in recent months. Certain developments could create challenges for global data sharing, although at this point any discussion of impact remains highly speculative. For example, in April 2018 the New York Times reported that the Trump administration is considering limiting the access of Chinese researchers to US technologies, based on national security and economic competitiveness concerns. According to the report, “[t]he exact types of projects that would be subject to restrictions are unclear, but the measures could clamp down on collaboration in advanced materials, software and other technologies at the heart of Beijing’s plan to dominate cutting-edge technologies like advanced microchips, artificial intelligence and electric cars” (Swanson and Bradsher 2018). Genomic data and tools for analysis are not mentioned, but some Chinese companies are exploring ways to employ artificial intelligence in the analysis of large collections of genomic or other health-related data. One of these companies, iCarbonX, recently made an investment in excess of $100 million in PatientsLikeMe, based in Cambridge, Massachusetts (PatientsLikeMe 2017). PatientsLikeMe holds health-related data for more than 500,000 people. It is unclear whether there is a plan for iCarbonX to gain access to this data in exchange for its investment.

One modest change, distinct from these larger national security and international trade concerns, would be consistent attention to global data sharing in consents. Given evidence of public concerns, there is a normative and, likely, legal case for disclosing the possibility that genomic data will be transferred to third countries as part of the informed consent process. Indeed, one of the basic principles stated in the Consent Policy developed by the Global Alliance for Genomics and Health is that “[d]ata donors have right to not participate in international data sharing” (Global Alliance 2015). The Public Population Project in Genomics and Society (P3G) and International Policy interoperability and data Access Clearinghouse (IPAC) have crafted a generic international data sharing prospective consent form template for the Global Alliance that includes statements about international aspects, including: “Your data will be used for international research and may be moved and stored [in controlled-access databases meeting international security and safety standards] in different countries… Your data will be shared with other researchers around the world and used in future biomedical research projects after ethics approval” (Global Alliance 2018). In light of public worries, the inclusion of information about safeguards and oversight is warranted. Furthermore, in the context of informed consent and general communications with the public, these kinds of disclosures should be complemented by information about the benefits of international data sharing, which provide the motivation for work to build a global medical information commons.

Finally, there are a number of developments pushing in the direction of a HIPAA update. Section 2063 of the Act directed DHHS to clarify requirements for authorizations for the use of PHI in future research (21st Century Cures 2016; OCR 2017). That section of the Act also directed DHHS to convene a working group to consider whether HIPAA should be modified to permit greater use of PHI for research purposes. In February 2017, the National Committee on Vital and Health Statistics sent a letter to the Secretary of DHHS recommending development of guidance to enhance the protection of privacy in the management of de-identified data (e.g., through business associate agreements) and greater transparency regarding distribution of de-identified and limited data sets (e.g., investigation of the feasibility of tracking such disclosures and including them in responses to data subjects’ requests for accountings of disclosures) (Stead et al. 2017). And, in the wake of the Cambridge Analytica scandal, commentators are giving serious consideration to the possible adoption of a general rule covering all personal data. A recent article by Cohen and Mello suggests three goals or guides for regulatory reform: avoiding undue burdens on the research and public health enterprises, giving individuals agency over their personal information “to the greatest extent commensurable with the first goal,” and holding data users accountable for violations (2018). The European General Data Protection Regulation has been put forward as a possible model for federal legislation addressing HIPAA’s limitations (Cohen and Mello 2018; Butterworth 2018), although such a move would have to counter a general antipathy in the US toward following rather than leading.

## Footnotes

1 A separate set of regulations contains the human subject protections that apply to clinical investigations regulated by, or for applications for research or marketing permits for products regulated by, the US Food and Drug Administration. The provisions are generally similar to those contained in the Common Rule, and Sect. 3023 of the Act requires harmonization of the two bodies of regulation, to the extent practicable, within 3 years.

2 Although the focus here is on US law and regulation at the federal level, at least one state has a genetic privacy law with an exception framed in similar terms. The Arizona law contains a general prohibition on disclosure of information derived from genetic testing; one exception applies if the research is conducted pursuant to applicable federal or state laws and regulations governing clinical and biological research. The Arizona law, like many other state laws, also allows disclosure for use in research if the information is first de-identified.
